# A Calcium Mediated Mechanism Coordinating Vascular Smooth Muscle Cell Adhesion During KCl Activation

**DOI:** 10.3389/fphys.2018.01810

**Published:** 2018-12-18

**Authors:** Huang Huang, Zhe Sun, Michael A. Hill, Gerald A. Meininger

**Affiliations:** Dalton Cardiovascular Research Center, Department of Medical Pharmacology and Physiology, University of Missouri, Columbia, MO, United States

**Keywords:** integrins, fibronectin, calcium, vascular smooth muscle, cell adhesion, cell stiffness, angiotensin II, atomic force microscopy

## Abstract

Efficient mechanotransduction in vascular smooth muscle cells (VSMCs) is intimately coupled to physical coupling of the cell to extracellular matrix proteins (ECM) by integrins. Integrin adhesion receptors are essential for normal vascular function and defective integrin signaling is associated with cardiovascular disease. However, less is known about the mechanism of integrin activation in VSMCs in relation to vasoregulation. Our laboratory previously demonstrated that the vasoconstrictor Angiotensin II increases VSMC stiffness in concert with enhanced adhesion to fibronectin (FN), indicating an important role for adhesion in contraction. However, the mechanism of this coordination remains to be clarified. In this study, intracellular Ca^2+^ ([Ca^2+^]_i_) was hypothesized to link integrin activation through inside-out signaling pathways leading to enhanced adhesion in response to AII. By using atomic force microscopy (AFM) with an anti-α_5_ antibody coated AFM probe, we confirmed that cell stiffness was increased by AII, while we observed no change in adhesion to an α_5_ integrin antibody. This indicated that increases in cell adhesion to FN induced by AII were occurring through an integrin activation process, as increased membrane integrin expression/receptor density would have been accompanied by increased adhesion to the anti-α_5_ antibody. Further studies were performed using either KCl or BAPTA-AM to modulate the level of [Ca^2+^]_i_. After KCl, VSMCs showed a rapid transient increase in cell stiffness as well as cell adhesion to FN, and these two events were synchronized with superimposed transient increases in the level of [Ca^2+^]_i,_ which was measured using the Ca^2+^ indicator, fluo-4. These relationships were unaffected in VSMCs pretreated with the myosin light chain kinase inhibitor, ML-7. In contrast, unstimulated VSMCs incubated with an intracellular calcium chelator, BAPTA-AM, showed reduced cell adhesion to FN as well the expected decrease in [Ca^2+^]_i_. These data suggest that in VSMCs, integrin activation is linked to signaling events tied to levels of [Ca^2+^]_i_ while being less dependent on events at the level of contractile protein activation. These findings provide additional evidence to support a role for adhesion in VSMC contraction and suggest that following cell contractile activation, that adhesion may be regulated in tandem with the contractile event.

## Introduction

The biomechanical properties of blood vessels are important determinants of cardiovascular function, and changes in these properties are characteristic of many cardiovascular diseases including hypertension, stroke, aneurysms, atherosclerosis and heart disease (Evans et al., 2016). The mechanical properties of the blood vessel wall are a function of the various wall components and their organization. Therefore, the stiffness of vascular tissue is resulted from both the composition and organization of the extracellular matrix (ECM) as well as the cells in the vascular wall which are interconnected by cell-matrix and cell-cell contacts (Saphirstein et al., 2013). It is commonly accepted that changes in the ECM are the dominate influence on vascular stiffness and that changes in collagen and elastin content and organization are of major importance (Dinardo et al., 2014; Xu and Shi, 2014). In addition to the ECM, it has been recently established that vascular smooth muscle cells (VSMCs), as the main cell type within the vascular wall, can exhibit increased stiffness that impacts the stiffness of the blood vessel wall. For example, Sehgel et al. (2013) found increased elastic stiffness of VSMCs in spontaneously hypertensive rats, and Zhu et al. (2012) have demonstrated higher stiffness in VSMCs from old monkeys compared to young monkeys. Cytoskeletal proteins and the contractile state have been associated with cell stiffness in VSMCs (Hong et al., 2012, 2014; Lim et al., 2012). In these studies, VSMC adhesion to ECM proteins was also shown to be enhanced in stiffer VSMCs. Fibronectin (FN) is a major ECM protein that interacts with integrin receptors on VSMCs and it is reported to accumulate in diseased artery walls (Pickering et al., 2000). Of importance, the α_5_β_1_ integrin is recognized as a major FN receptor and plays an important role in VSMCs mechanoreception linking it to vascular regulation (Francis et al., 2002; Sun et al., 2005).

Integrins, as heterodimeric membrane receptors, composed of two non-covalent associated transmembrane subunits –α and -β, provide an important physical connection between the ECM and the cytoskeleton, maintaining a dynamic adhesion between the cell and its microenvironment. This unique feature positioning of integrins makes them ideally situated for bi-directionally transmitting and conducting mechanical signals through both inside-out and outside-in mechanisms. Integrins are known to regulate cell differentiation, proliferation, and survival through controlling critical intracellular signals (Lee et al., 2010; Paszek et al., 2014; Sarrazy et al., 2014). Importantly, evidence exists to support an important role for integrins in regulating vascular function (Mogford et al., 1996; D’Angelo et al., 1997; Platts et al., 1998). Among the multitude of integrin receptors identified on the surface of VSMCs, integrin α_5_β_1_ acts as the dominant FN receptor (Pickering et al., 2000; Sun et al., 2005; Trache et al., 2005). In previous work from our laboratory, Hong et al. demonstrated the existence of coordination between cell stiffness and cell adhesion of VSMCs to FN as they respond to the contractile vasoactive agonist Angiotensin II (AII) or to the vasodilators adenosine and nitric oxide (Hong et al., 2012, 2015). This coordination of adhesion with the contractile state was suggested to be regulated by changes in integrin behavior (Hong et al., 2012). The function of integrin can be controlled by both ‘outside-in’ signaling and “inside-out signaling” (Banno and Ginsberg, 2008). In the case of VSMC activation by soluble vasoactive agonists, it is likely that changes in the integrin activation state are occurring through an ‘inside-out activation’ process from within the cell. Such changes in integrin activation occur through conformational changes in the extracellular integrin head domains in which they can switch reversibly from a low-affinity conformation to a high-affinity active conformation (Calderwood, 2004). Integrin α_5_β_1_ has been observed to undergo conformational changes in focal adhesions (Askari et al., 2010), and these changes have been demonstrated to occur with alterations in cytoskeletal tension (Friedland et al., 2009; Kong et al., 2009). However, the mechanism underlying the changes in integrin activation in VSMCs in response to vasoactive-agonists requires further study.

In this study, we hypothesized that the coordination of cell stiffness and cell adhesion to FN of VSMCs is linked to changes in intracellular calcium ([Ca^2+^]_i_) which is known to activate the contractile process. Therefore, the enhanced adhesion in response to AII may result from integrin activation through an ‘inside-out’ signaling pathway linked to an increase in [Ca^2+^]_i_. To address this hypothesis, we utilized atomic force microscopy (AFM) combined with confocal microscopy. Using AFM it is possible to detect and apply mechanical forces at the nano- and pico-newton ranges, providing a powerful tool for investigating biomechanical properties of VSMCs (Smith et al., 2005) and for simultaneously measuring changes in cell adhesion molecules (Baumgartner et al., 2000; Kong et al., 2009; Carvalho and Santos, 2012). In this study, AFM was used for real-time dynamic observations of cell stiffness and adhesion and correlate them with [Ca^2+^]_i,_ measured using the calcium indicator fluo-4. We show that cell stiffness and cell adhesion to FN change directionally with [Ca^2+^]_i_. We also observed that increased cell stiffness and adhesion to FN was not abolished by treatment with a myosin light chain kinase (MLCK) inhibitor. Thus, the changes in cell stiffness and adhesion appear to occur by a signaling pathway parallel to but not downstream of contraction.

## Materials and Methods

### Reagents and Materials Applied

For all studies described, human plasma fibronectin (FN), 1,2-bis(o-aminophenoxy)ethane-N,N,N′,N′-tetraacetic acid (acetoxymethyl ester) (BAPTA-AM), and fluo 4-AM were purchased from Invitrogen (Carlsbad, CA, United States), Bovine Serum Albumin (BSA), KCl, AII were purchased from Sigma (St. Louis, MO, United States). Antibodies to integrin α5 (CD49e, cat: 553350), phalloidin 488 were purchased from Abcam (Cambridge, United Kingdom). For all cell isolations, dithioerythritol (Dith), papain, collagenase, soybean trypsin inhibitor, and elastase were purchased from Sigma (St. Louis, MO, United States). For studies using atomic force microscopy (AFM), a Bioscope II AFM system (Bruker, Santa Barbara, CA, United States) was mounted on a Fluoview confocal microscope (Olympus, Thornwood, NY, United States). AFM data were collected and analyzed using Nanoscope (Bruker, Santa Barbara, CA, United States) and Matlab (MathWorks, Natick, MA, United States) software. Confocal images were analyzed by using Image-Pro Plus software (Media Cybernetics, Carlsbad, CA, United States).

### Cell Preparation

#### Animal and Tissue Preparation

Male Sprague-Dawley rats (200–300 g) were used in this study and were maintained in accordance with the protocol of the Guide for the Care and Use of Laboratory Animals (NIH 83-23, revised 1996). The animal protocol was reviewed and approved by the Laboratory Animal Use Committee (ACUC) of the University of Missouri. Animals were housed individually in a 12:12 light-dark cycle with free access to food and water. Arterioles were isolated as described in previous work with minor modification (Hong et al., 2012). Briefly, rats were anesthetized by intraperitoneal injection of pentobarbital sodium (Lundbeck, Inc., Deerfield, IL, United States) at 0.1 g/kg. After the anesthesia was verified by lack of spinal reflex, the cremaster muscle was removed through a scrotal surgical incision while rats remained under anesthesia. The tissue was immediately placed in cold (4°C), Ringer’s buffer (composed of 145 μM NaCl, 4.7 μM KCl, 2.0 μM CaCl_2_-2H_2_O, 1.0 μM MgSO_4_-7H_2_0, 1.2 μM NaH_2_PO_4_-H_2_O, 0.02 μM EDTA-2H_2_O, 3.0 MOPS, 5 μM Glucose, 2.0 μM Pyruvic Acid). Vessels and cell isolations were performed in solution based on Ringer’s buffer. Anesthetized rats were euthanized by intracardiac injection of saturated KCl solution (3 ml). The cremaster muscles were then pinned flat onto a silicone rubber pad in a bath of cold Ringer’s buffer. With the aid of a stereomicroscope (Olympus SZX10, Thornwood, NY, United States), long segments of the first and second order cremasteric feed arterioles vessel were dissected and isolated from surrounding skeletal muscle tissue. The isolated arteriole segments were removed and cut into small pieces (∼2–3 μm) and were placed into culture dishes (35 μm) with Ringer’s buffer in room temperature, and subsequently processed to isolate vascular smooth muscle cells (VSMCs).

#### VSMCs Isolation and Culture

Vascular smooth muscle cells were enzymatically isolated using previously described methods (Jackson et al., 1997). Briefly, arteriolar segments were transferred to a glass culture tube (10 μm × 75 μm) that contained 1 ml of the first cell dissociation solution that Ringer’s buffer included 27 U/ml papain and 1 mg/ml dithioerythritol. The segments were incubated at 37°C for 30 min without agitation. Following this incubation, the first cell dissociation solution was carefully decanted and replaced with 1 ml of second cell dissociation solution consisting of Ringer’s buffer and 0.5 U/ml collagenase, 75 U/ml elastase, and 1 mg/ml soybean trypsin inhibitor. The segments were further incubated for 12–15 min at 37°C. At the end of the incubation period, the dissociation solution was carefully decanted with that not disturb the arteriole segment. Arteriole segments were then very carefully and gently washed twice by adding 1 ml Ringer’s buffer, followed by 1 ml serum-free supplemented Dulbecco’s modified Eagle’s medium (DMEM)/F12 (Invitrogen, Carlsbad, CA, United States). The vessels segments were pipetted with a glass pipette (1 ml) for about 20–30 times to disperse individual cells from the tissue. The solution containing the dispersed VSMCs was then decanted into 60 μm tissue culture dishes (Corning incorporated, Corning, NY, United States), and maintained under culture conditions in a humidified incubator with 5% CO_2_, at 37°C, in DMEM supplemented with 20% fetal bovine serum (FBS, ATLANTA Biologicals, Lawrenceville, GA, United States), 10 mM HEPES (Sigma, St. Louis, MO, United States), 1 mM sodium pyruvate (Sigma, St. Louis, MO, United States), 2 μM L-glutamine (Sigma, St. Louis, MO, United States), 100 U/ml penicillin (Sigma, St. Louis, MO, United States), and 100 g/ml streptomycin (Sigma, St. Louis, MO, United States). After 48 h incubation, the culture medium was changed to DMEM with 10% FBS. The cells used in all experiments were maintained under these conditions for 5–10 days without passage. Prior to an experiment, the VSMCs were serum starved overnight.

### Force Spectroscopy Measurement

#### Bio-Functionalized AFM Probes

The AFM probe tip was modified by gluing a 5 μm diameter glass bead (Structure Probe Inc., West Chester, PA, United States) to the tip of cantilever (MLCT-O10, Bruker Corp., Santa Barbara, CA, United States). The bead tip was then coated with FN or anti-α_5_ antibody using the protocol previously used in our laboratory (Sun et al., 2005). Briefly, the bead was first glued to a clean AFM probe and then FN was attached to bead by a cross-linker. Before gluing the glass bead to the cantilever tip, each cantilever was calibrated before a given experiment using thermal noise amplitude analysis. Cantilevers were then cleaned first by rinsing in 100% ethanol twice followed by rinsing in acetone for three times. The cantilever was dipped in glue (Progressive Epoxy Polymers Inc., United States) and then brought into contact with a glass bead mixture in solution that had been smeared on a glass slide. After bead attachment, AFM probes were stored for 48 h to allow bead glue to cure. The AFM probe with attached bead was then processed to bio-coat the bead tip. The tip was immersed for 5 min in 10 mM Polyethylene glycol (PEG, Sigma, St. Louis, MO, United States), which acted as a cross-linker used for attachment of proteins of interest to the bead. This was followed by washing with distilled water four times, and incubating in a solution of FN (0.25 mg/ml) or anti-α_5_ antibody (1 mg/ml) for 5 min, followed by cleaning four times with Phosphate-Buffered Saline (PBS) (1.05 μM KH_2_P0_4_, 155.17 μM NaCl, 2.96 μM Na_2_HPO_4_-7H_2_O). For all experiments, the AFM spring constants were assumed to be unchanged after protein labeling (Lehenkari and Horton, 1999).

#### Nano-Indentation Protocol of AFM

The biomechanical properties of the VSMCs were measured using a Bioscope II AFM system (Bruker, Santa Barbara, CA, United States) that was mounted on an IX81 OLYMPUS inverted microscope (Olympus Inc., NY, United States). AFM measurements were carried out at room temperature in a 1% HEPES supplemented DMEM without antibiotics. For each experiment, cells were randomly selected and the functionalized AFM probe was repeatedly brought into contact with and retract from the cell surface. This cyclic approach/retraction cycle was performed at a site midway between the nucleus and cell margin for frequency of 0.1 Hz and ramp size of 1000 nm. 60 force curves were collected over 10 min for a control (pre-drug) period followed by 240 force curves over 40 min for the post-drug condition. To minimalize the machine drift, after the probe was initially submerged in the cell bath, the AFM-microscope system was thermally equilibrated for 1 h. The analysis of force curves was processed by a custom analysis program written in MATLAB.

#### Measurement of Biomechanical Properties With AFM

For the nano-indentation protocol, the stiffness measured is an estimation of Young’s modulus of elasticity (E-modulus) for the cell cortex, reflecting the cell’s viscoelastic properties. A length of approximately 100–300 nm of the AFM indentation curve, following the initial point of contact, was fitted to a modified Hertz Model, which was used to assess the VSMC elasticity.

$$F=\frac{4}{3}\cdot \frac{\sqrt{r}\cdot E}{1-v^{2}}\cdot \delta^{3/2}$$

Here, F is the force applied by the AFM probe on the cell surface; *E* is the E-modulus; *v* is the Poisson ratio (assumed as 0.5); *r* is the radius of spherical AFM tip; δ is the indentation depth into the cell membrane. Rupture force, also referred to here as adhesion force between FN and integrin adhesion complexes, was the product of rupture height and cantilever spring constant, measured from the retraction curve (Hong et al., 2012).

#### AFM Contact Mode Imaging

To obtain a topographical cell image, the AFM tip was placed on the cell surface and then using scanning mode was moved horizontally along the cell surface while applying a constant force (500 – 800 pN) to the cell surface. Scanned images were 100 μm × 100 μm at the digital density of 512 pixels × 512 pixels. A stylus-type AFM probe (model MLCT-C, k = 15 pN nm^-1^, Bruker, Santa Barbara, CA, United States) was used to perform the cell surface scanning at 0.15 Hz frequency at room temperature. Height and deflection images were collected with Bioscope software and analyzed using Nanoscope software.

### Measurement of Intracellular Calcium

#### Fluo-4 AM Loading of VSMCs

Intracellular calcium was measured by imaging fluo-4 AM. Cells cultured on glass-bottomed tissue culture dishes (Corning incorporated, Corning, NY, United States) were rinsed by loading buffer (150 μM NaCl, 5 mM KCl, 1 μM MgCl, 10 μM glucose, and 20 μM HEPES, pH 7.4) twice and then bathed with fluo 4-AM solution (2.5 μM, Invitrogen Corp., Carlsbad, CA, United States), which is dissolved in loading buffer supplemented with 2% BSA and 0.01 % Pluronic F-127 (BASF) protected for light for 25 min at room temperature on a rolling plate. Cells were then washed twice using loading buffer and incubated in serum-free DMEM at 30°C for 20 min to allow the de-esterification.

#### BAPTA-AM Loading of VSMCs

Cells were cultured on glass-bottomed tissue culture dishes. Cells were rinsed twice with loading buffer followed by a 25-min incubation with 20 μM BAPTA-AM (Invitrogen, Carlsbad, CA, United States) dissolved in loading buffer supplemented with 2% BSA and 0.01 % Pluronic F-127 (BASF, Sigma, St Louis, MO, United States) on a rolling plate agitator at room temperature. After loading, cells were then washed twice with loading buffer and incubated in serum-free DMEM for 20 min at 30°C to allow the de-esterification.

### Fluorescence Imaging

To assess the correlation between cell stiffness and adhesion with intracellular calcium levels, we performed calcium imaging and made AFM measurements simultaneously. Calcium fluorescence images were continuously recorded 20 s before pulling the AFM until 20 s after pulling. A confocal microscope (FV1000) was used to imagine Fluo-4-loaded VSMCs with laser of 488 nm. Fluorescence used for all imaging experiments was collected at 510∼550 nm ×60 oil immersion objective. Fluo-4 images were analyzed using Fluoview software.

### Immunocytochemistry and Confocal Microscopy

Prior to immunocytochemistry, VSMCs were serum-starved overnight. After treatment under different conditions (AII treated at 10^-6^M for 30 min versus PBS exposure for 30 min, as a control), cells were washed three times with PBS followed by fixation with 2% paraformaldehyde for 30 min at room temperature. Cells were then quenched with glycine buffer (0.1 μM glycine) three times for 10 min. After quenching, cells were bathed with primary antibody overnight at 4°C, 1:100 diluted in labeling buffer (150 μM NaCl, 15 μM Na_3_C_6_H_5_O_7_, 0.05% Triton X-100, 2% BSA). Cells were then rinsed (six times) with cold washing buffer (150 μM NaCl, 15 μM Na_3_C_6_H_5_O_7_, 0.05% Triton X-100), followed by secondary antibody incubation (Cy5-labeled or Alex 488-conjugated phalloidin), 1:200 diluted in labeling buffer, for 2 h in a dark environment on a rolling plate at room temperature. The cells were washed again with cold washing buffer and visualized with a confocal microscope. Laser of 647 and 488 nm were used, respectively, for Cy5 label and phalloidin. A through-focus image set was collected for each cell with a z step interval of 0.2 μm.

### Statistical Analysis

All data are reported as mean ± SEM. Statistical differences for comparisons of pre-drug controls and post-drug treatments were analyzed with paired *t*-test. ANOVA was used for between-group analysis. All statistical analyses were performed using open source software SciDAVis and StatPlus (AnalystSoft Inc., CA, United States). Differences were considered significant at *p* ≤ 0.05.

## Results

### Membrane Expression of Integrin α_5_ Following Angiotensin II (AII) Treatment

**FIGURE 1:**
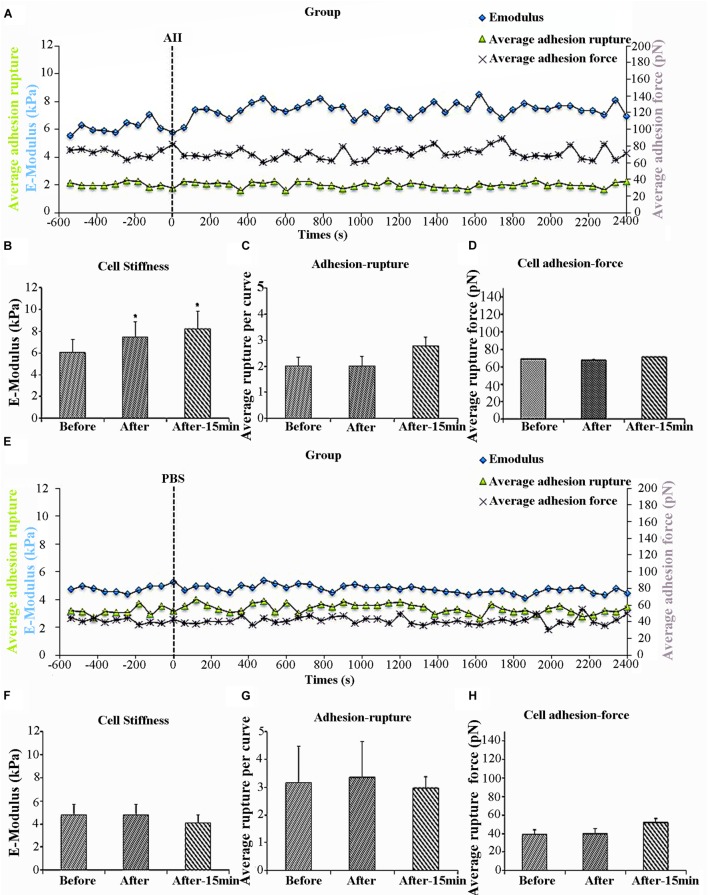
Angiotensin II (AII) increased vascular smooth muscle cells (VSMCs) stiffness but had no effect on cell adhesion to anti-α_5_ antibody. Group average real-time recordings of cell stiffness (blue square), rupture events (green triangle), and rupture force (purple cross) for AII (*n* = 5*^t^*) treatment **(A)** and the addition of PBS (*n* = 10) **(E)**. Average elastic modulus summed across all or 15 min of time points for the group of VSMCs before and after AII (10^-6^ M) treatment **(B)** (*n* = 5, ^∗^*p* < 0.05) and PBS addition **(F)** (*n* = 10, ^∗^*p* < 0.05). Average rupture numbers per curve summed across all or 15 min of time points for the group of VSMCs before and after addition of AII **(C)** (*n* = 5, ^∗^*p* < 0.05) and PBS **(G)** (*n* = 10, ^∗^*p* < 0.05). **(D)** Average rupture force summed across all or 15 min of time points for the group of VSMCs before and after addition of AII **(D)** (*n* = 5, ^∗^*p* < 0.05) and PBS **(H)** (*n* = 10, ^∗^*p <* 0.05). Data were collected by integrin α5 antibody coated AFM tip at 0.1 Hz of indentation frequency and are presented as mean ± SEM. *^t^*As detailed in the text while eight cells were treated with AII analysis was limited to the five cells responding with the characteristic increase in cell stiffness as observed in previous studies.

Previous work in our laboratory has shown that AII increases VSMCs stiffness as well as adhesion to FN (Hong et al., 2012, 2014). Specificity of FN adhesion to α_5_β_1_-integrin on VSMCs was confirmed by demonstrating that the adhesion was inhibited with function-blocking antibodies to α_5_ or β_1_ integrins (Sun et al., 2005). To determine whether the increased cell adhesion during AII treatment was due to enhanced membrane expression of integrin α_5_β_1_, we used AFM probes coated with anti-α_5_ antibody, since β_1_ integrin would also be present in other integrin dimers. We hypothesized that if AII was increasing the number of α_5_ integrins on the cell surface, then adhesion to the antibody-coated probes would increase. In these experiments, 8 cells were treated with AII and of these five responded with the characteristic increase in cell stiffness whereas 3 of the cells did not respond. Only responsive cells were analyzed. The increase in cell stiffness (blue circle) was gradual after AII exposure and reached a plateau after approximately 15 min, which is consistent with previous reports from our laboratory (Figure 1A). Before treatment, average cell stiffness was 6.04 ± 0.26 kPa, and which increased to 7.47 ± 0.21 kPa after AII representing a statistically significant increase by 24% (Figure 1B). As a control, VSMCs were treated with PBS which showed no effect on cell stiffness (Figures 1E,F).

Cell adhesion was analyzed in the AII responsive cells and was quantified as the number of ruptures per curve (Figure 1A, green triangles) and the total rupture force (Figure 1A, purple crosses). After AII treatment, cell adhesion to the anti-α_5_ antibody, measured as rupture number per curve (Figure 1C), was not significantly increased. In addition, rupture force showed no observable increase with AII (Figure 1D). PBS-treated cells also showed no effect on average rupture number per curve or total rupture force (Figures 1E,G,H).

**FIGURE 2:**
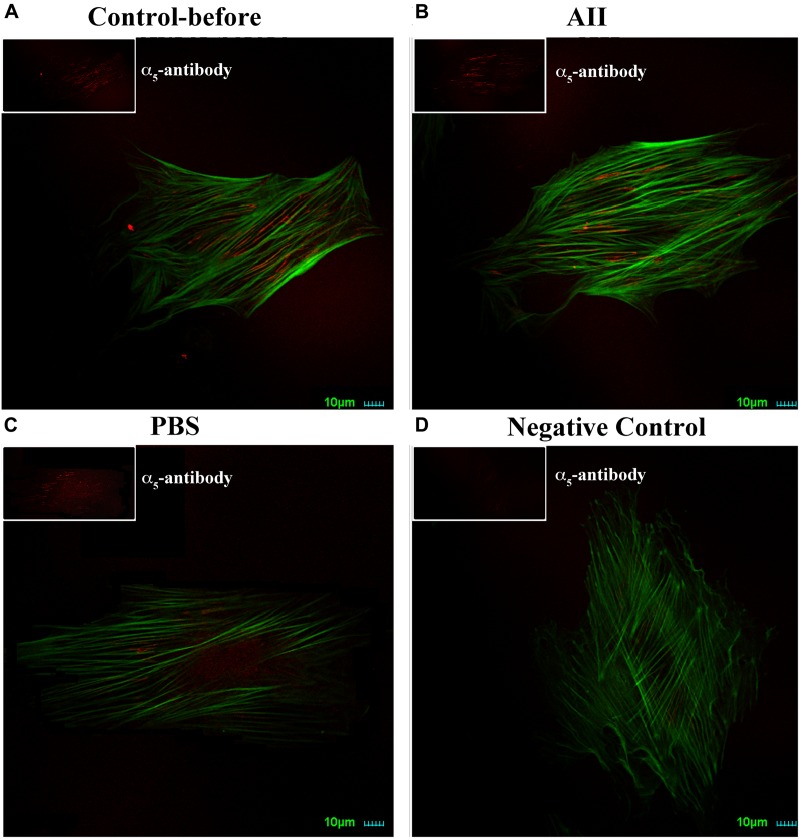
AII has no effect on expression of integrin α_5_ in VSMCs. Representative images for six cells studied under each condition. Note should be made that it cannot be excluded that some of the cells were non-responders as these cells were not subjected to AFM functional studies for technical reasons. **(A–D)** Immunostaining of integrin α_5_ (red) and labeling with phalloidin (green) on VSMCs before treatment **(A)**, after addition of AII (10^-6^ M) for 30 min **(B)** and PBS **(C)**. **(D)** Is negative control without anti-integrin α_5_ as the primary antibody. Scale bars represent 10 μm.

As a further test to confirm a lack of change in expression of integrin α_5_, we also performed immunostaining of α_5_ on VSMCs. Cells were co-labeled with phalloidin to visualize cell stress fibers. Distribution of α_5_ integrin was diffusely present on the apical surface of VSMCs observed by immunofluorescence confocal microscopy (Figure 2A). After treatment with AII, there were no observable changes of α_5_ distribution on the cell surface or intensity of labeling (Figure 2B). As the control group, PBS treatment also had no effect on α_5_ distribution or labeling intensity (Figure 2C). Collectively, these data suggest that AII-induced increases in VSMCs adhesion to FN does not result from enhanced membrane expression of integrin α_5_. Rather, these data are consistent with an effect of AII to increase integrin activation. As caveat to the experiments shown in Figure 2 it cannot be excluded that some of these cells were non-responders (as in Figure 1) as these cells were not subjected to AFM functional studies for technical reasons.

### Effect of KCl Treatment on VSMC Stiffness and Adhesion to FN

**FIGURE 3:**
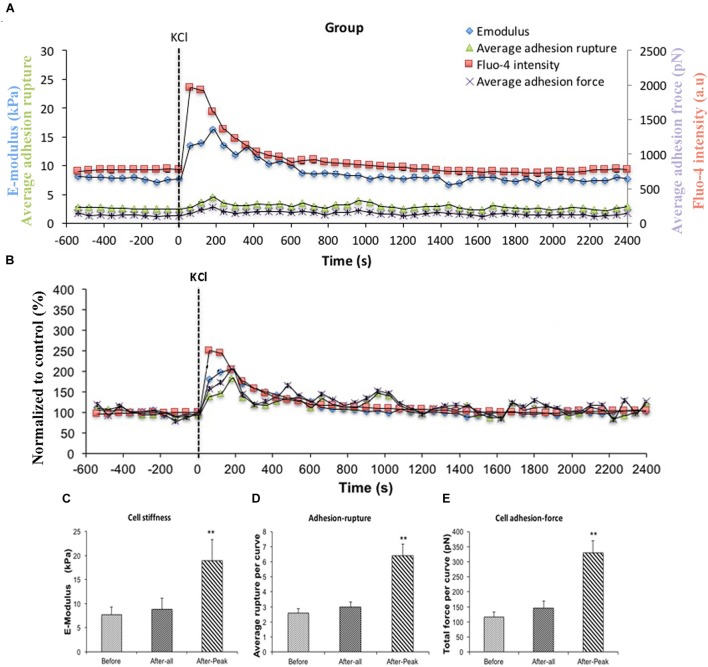
KCl transiently enhanced cell stiffness and cell adhesion to Fibronectin (FN) as well as intracellular calcium level ([Ca^2+^]_i_). All cells responded to KCl treatment. **(A)** Group average real-time recordings of [Ca^2+^]_i_ (red), cell stiffness (blue), rupture events (green), and rupture force (purple) before and after addition of KCl (*n* = 10). **(B)** Group recordings of [Ca^2+^]_i_ (red), cell stiffness (blue), rupture events (green), and rupture force (purple) normalized to average summed all time points of before-treatment (*n* = 10). **(C)** Average elastic modulus summed across all of time points and peak response for the group of VSMCs before and after addition of KCl (60 μM) (*n* = 10, ^∗∗^*p* < 0.01). **(D)** Average rupture numbers per curve summed across all of the time points and peak response for the group of VSMCs before and after addition of KCl (*n* = 10, ^∗∗^*p* < 0.01). **(E)** Average rupture force summed across all of the time points and peak response for the group of VSMCs before and after addition of KCl (*n* = 10, ^∗∗^*p* < 0.01). Data were collected by FN-coated AFM tip at 0.1 Hz of indentation frequency and are presented as mean ± SEM.

AII is known to act on the AT1R and to cause increases in intracellular Ca^2+^ ([Ca^2+^]_i_) in VSMCs (Touyz and Schiffrin, 2000; Simo-Cheyou et al., 2017). To test the hypothesis that [Ca^2+^]_i_ was playing a role in the enhanced adhesion to FN and increase in cell stiffness we utilized KCl (60 μM). KCl is a non-receptor agonist which increases levels of [Ca^2+^]_i_ secondary to cell depolarization. In these experiments, FN (0.25 mg/ml) coated AFM probes were used to measure cell stiffness and adhesion response to KCl. In this protocol, all 10 cells studied showed a response to KCl treatment. Before treatment, average cell stiffness was 7.64 ± 1.65 kPa, and application of KCl induced a rapid transient and significant increase in cell stiffness to 18.87 ± 4.39 kPa (Figure 3A, blue circle) representing a 246% increase (Figure 3B, blue square) (Figure 3C). During the period when cell stiffness was transiently elevated we observed that cell adhesion to FN was also transiently increased. The increase in adhesion was apparent as a significant increase (248%) in the numbers of ruptures per curve (Figures 3A,D, green triangle) and increase in total adhesion force (284%) (Figures 3A,E, purple cross).

### Involvement of [Ca^2+^]_i_ as a Mediator of Changes in Cell Stiffness and Adhesion

**FIGURE 4:**
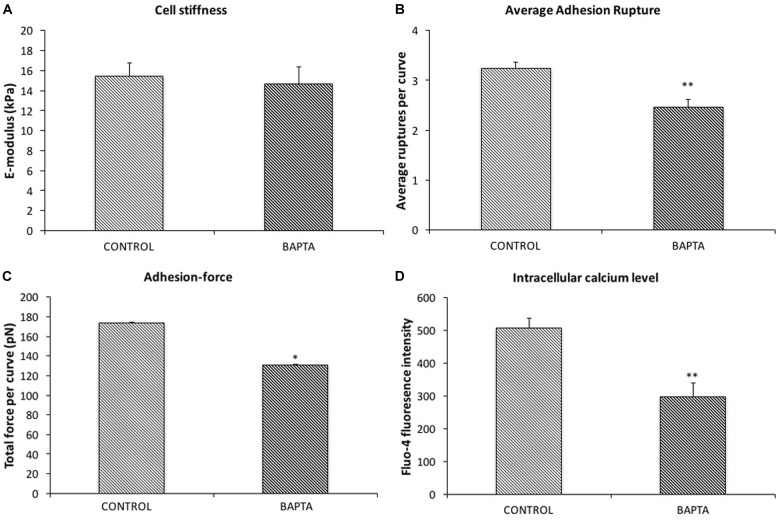
BAPTA treatment decreased cell adhesion to FN but has no effect on cell stiffness. **(A)** The average elastic modulus for the group of VSMCs with and without BAPTA-treatment (20 μM). **(B)** Average rupture events per curve for the group of VSMCs with and without BAPTA treatment (*n* = 10, ^∗∗^*p* < 0.01). **(C)** Average rupture for the group of VSMCs with and without addition of BAPTA (*n* = 10, ^∗∗^*p* < 0.01). **(D)** Level of [Ca^2+^]_i_ for the group of VSMCs with and without addition of BAPTA (*n* = 10, ^∗∗^*p* < 0.01). Data were collected by FN-coated AFM on randomly selected five cells, each cell was recorded 2 min at 0.1 Hz of indentation frequency. Data are presented as mean ± SEM.

To confirm Ca^2+^ was changing following KCl treatment, VSMCs were loaded with the fluorescent calcium indicator fluo-4. [Ca^2+^]_i_ measurements were obtained while simultaneously performing AFM to measure cell stiffness and adhesion to FN. A transient increase in levels of [Ca^2+^]_i_ induced by KCl that correlated with the increased cell stiffness, as well as the increases in adhesion ruptures and total adhesion force, which is cumulative ‘pull-off’ force measured for each curve (Figures 3A,B, red square). It was noted that [Ca^2+^]_i_ levels reached maximum levels immediately preceding the changes in cell stiffness and cell adhesion. These data suggest that calcium-related signaling is linked to the observed changes in cell stiffness and adhesion. As a further test of the involvement of [Ca^2+^]_i_ as a mediator of the observed changes in stiffness and adhesion, resting VSMCs were treated with the calcium chelator, BAPTA to remove Ca^2+^ from the intracellular compartment. BAPTA was applied under basal or resting conditions (i.e., in the absence of AII or KCl) as our earlier studies have shown that cells have a baseline level of stiffness that can be decreased by treatment with agents that cause relaxation (for example adenosine and sodium nitroprusside) (Hong et al., 2014, 2015). As shown in Figure 4A, BAPTA-treated groups of cells showed slightly decreased stiffness, however, this reduction was not statistically significant compared to cells of PBS-treated groups. However, average adhesion ruptures significantly declined 24% (*p* < 0.01) in BAPTA-loaded cells (Figure 4B) compared to the control group. BAPTA treated cells also showed a significant reduction in total adhesion force, which decreased 25% (Figure 4C). Fluo-4 was loaded VSMCs to measure [Ca^2+^]_i_ in BAPTA treatment, BAPTA-loaded cells showed significantly decreasing [Ca^2+^]_i_ compared to the PBS-treated control group at the p level of 0.01 (Figure 4D). Thus, these data further suggest [Ca^2+^]_i_ plays a role in altering integrin adhesion.

### Effects of Inhibition of Myosin Light Chain Kinase

**FIGURE 5:**
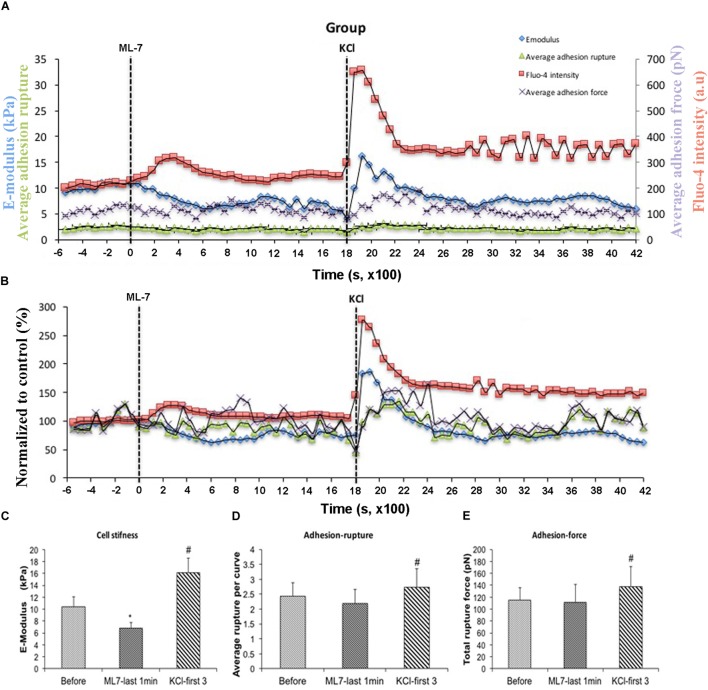
With pre-treatment of ML-7, KCl elevated cell stiffness, cell adhesion to FN as well as the level of [Ca^2+^]_i_. All cells responded to KCl treatment. **(A)** Group average real-time recordings of [Ca^2+^]_i_ (red), cell stiffness (blue), rupture events (green), and rupture force (purple) before and after addition of ML-7 and KCl (*n* = 10). **(B)** Group recordings of [Ca^2+^]_i_ (red), cell stiffness (blue), rupture events (green), and rupture force (purple) normalized to average summed all time points without treatment (*n* = 10). **(C)** Average elastic modulus summed across all time points for the group of VSMCs before treatment, time points of 15 min of ML-7 (15 μM) and first three time points after addition of KCl (60 μM) (*n* = 10, ^∗^*p* < 0.05 compared to no-treatment, ^#^*p* < 0.05 compared to ML-7). **(D)** Average rupture numbers per curve summed across all time points for the group of VSMCs before treatment, time points of 15 min of ML-7 (15 μM) and first three time points of after addition of KCl (60 μM) (*n* = 10, ^∗^*p* < 0.05 compared to no-treatment, ^#^*p* < 0.05 compared to ML-7). **(E)** Average rupture force summed across all of time points for the group of VSMCs before treatment, following 15 min pre-incubation with ML-7 (15 μM) and first three time points of after addition of KCl (60 μM) (*n* = 10, ^∗^*p* < 0.05 compared to no-treatment, ^#^*p* < 0.05 compared to ML-7). Data were collected by FN-coated AFM at 0.1 Hz of indentation frequency and are presented as mean ± SEM.

**FIGURE 6:**
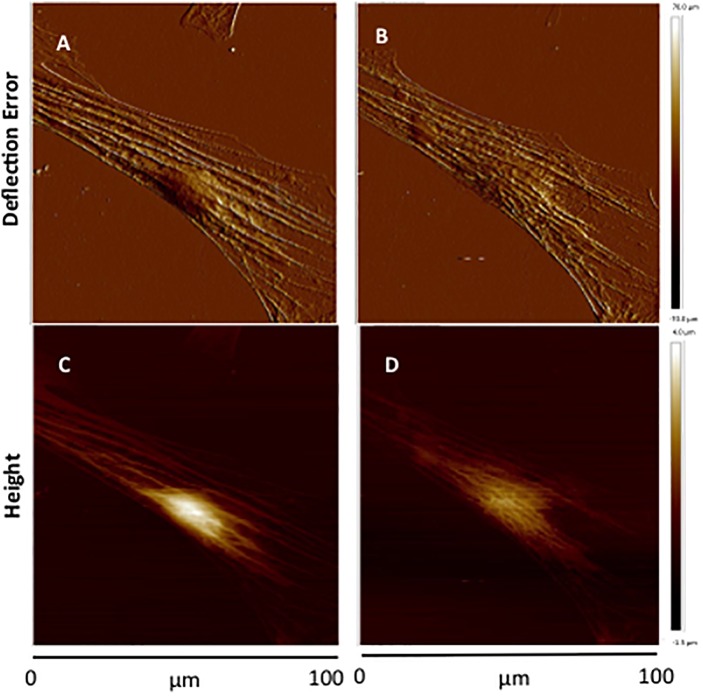
Representative contact-mode images of VSMCs treated with ML-7. **(A,B)** Deflection error images of single cultured VSMC before treatment **(A)** and after ML-7 (15 μM, 45 min) treatment **(B)**. **(C,D)** Representative height images of single cultured VSMC of before treatment **(C)** and after addition of ML-7 (15 μM) **(D)**. Group data for maximal cell height were 3.2 ± 0.2 μm before and 2.6 ± 0.2 μm after ML-7 (*n* = 9; *p* < 0.01).

[Ca^2+^]_i_ elevation leads to VSMC contraction through activation of myosin light chain kinase (MLCK) via calcium–calmodulin complex (Misárková et al., 2016). To further investigate the role of [Ca^2+^]_i_ in cell stiffness and adhesion to FN, VSMCs were treated with ML-7, a myosin light chain kinase inhibitor prior to treatment with KCl. [Ca^2+^]_i_ was also measured using fluo-4. We have previously shown that ML-7 inhibits depolarization-induced induced smooth muscle contraction while not significantly altering the associated intracellular Ca^2+^ signal increases (Zou et al., 1995). Following treatment with ML-7, VSMCs E-modulus was decreased to 35% (Figure 5B, blue diamond) consistent with a loss of contractile tone in the cells. These data were normalized to the pre-drug period and compared to the last 10 min of ML-7 treatment (Figure 5C) In contrast there were no significant effects on adhesion rupture (Figure 5D) or adhesion force (Figure 5E). [Ca^2+^]_i_ by ML-7 while KCl continued to cause a similar acute increase in Ca^2+^_i_ (compare Figure 5B with Figure 3B, Supplementary Figure S1). Compared to the pre-drug condition (Figure 6A), ML-7 treatment was associated with a loss of stress fibers or the large bands of actin cytoskeletal filaments underlying the cell membrane (Figure 6B). Topographic images of the VSMCs also revealed cell height decreased following ML-7 (Figures 6C,D); maximal cell height 3.2 ± 0.2 μm before and 2.6 ± 0.2 μm after ML-7 (*n* = 9; *p* < 0.01). Following application of KCl to the ML-7-treated cells (Figure 5) there were no observable changes in the effect of KCl on stiffness or adhesion as either rupture numbers or adhesion force. Overall, these results are consistent with an involvement of [Ca^2+^]_i_ in cortical cell stiffness and adhesion to FN. Furthermore these data suggest that increased cell stiffness and adhesion were not dependent on actin-myosin interaction.

## Discussion

Previously, Hong et al. (2012, 2015) have shown in VSMCs that vasoactive agonists induce coordinated changes in cell elasticity and integrin-mediated cell adhesion. That specifically, vasoconstrictors increase VSMC elastic modulus and simultaneously enhance cell adhesion whereas vasodilators have the opposite effect (Hong et al., 2012). In this study, we performed a series of experiments to examine the hypothesis that intracellular Ca^2+^ ([Ca^2+^]_i_) is a signaling link that plays a role in coordinating changes in cell adhesion that occur when contraction is activated. We first examined, whether contractile activation with AII would alter integrin expression or activation on the cell surface. Our results supported an integrin activation process which led to additional experiments designed to specifically probe the role intracellular Ca^2+^. KCl was used as a contractile agonist to elevate intracellular Ca^2+^, BAPTA was used to lower intracellular Ca^2+^ and ML-7 to explore the role of a Ca^2+^ dependent actin-myosin interaction. The ensuing discussion is organized in support of the concept that the regulation of ECM adhesion is linked to the contractile activation state of vascular smooth muscle. The results suggest this coordinated link is synchronized with [Ca^2+^]_i_. We tested the hypothesis that adhesion to the ECM protein FN would be enhanced with elevated [Ca^2+^]_i_ in VSMCs_,_ and that the change in adhesion would occur along with increases in VSMC cortical stiffness. The increase in cortical stiffness was assumed to act as an indication of changes in the state of VSMC contractile activation. As a corollary to this, we postulated that lower [Ca^2+^]_i_ would produce opposite results characterized by decreased adhesion to FN and a parallel reduction in VSMC cortical stiffness.

Real-time fluo-4 confocal image combined with AFM was used to record cell stiffness and adhesion to FN along with [Ca^2+^]_i_. We confirmed that increases in cell stiffness and adhesion to FN occurred in the presence of a rapid but transiently increase [Ca^2+^]_i_ that was induced by KCl. The results indicated a high degree of correlation between the increase in [Ca^2+^]_i_ and cell stiffness and adhesion. As the contraction of VSMC follows from increased [Ca^2+^]_i_, we inhibited the contractile interaction of actin and myosin with ML-7. By presumably preventing contractile filament interaction, we sought to determine whether the changes in cortical stiffness and cell adhesion were linked as a downstream event of the contractile process or occurred as a set parallel event coordinated with contraction through changes in [Ca^2+^]_i._ The results of these experiments indicated that the changes in cell stiffness and adhesion persisted in the presence of the inhibition of MLCK supporting the interpretation that that coordination with contraction is likely regulated by a pathway parallel to but not downstream of contraction. We then sought to lower [Ca^2+^]_i_ by incubation with the calcium chelator BAPTA. We reasoned that lowering [Ca^2+^]_i_ would not only inhibit contractile filament interaction but would also inhibit any parallel pathway dependent on [Ca^2+^]_i._ The results confirmed that lowering [Ca^2+^]_i_ reduced cellular adhesion to FN providing additional support for the conclusion that there is a parallel pathway activated upstream of the contractile pathway. It should be noted that BAPTA was applied under basal conditions (i.e., in the absence of added contractile agents) as our earlier studies have shown that cells have a basal level of stiffness which can be decreased by the addition of agents known to cause relaxation (e.g., Adenosine and nitroprusside) (Hong et al., 2014, 2015). While this approach was taken to examine the effects of Ca^2+^ depletion on inherent VSMC mechanical properties future studies could repeat this approach after contractile activation in response to exogenous agents.

The cytoskeleton of VSMC provides a structural framework that determines cell shape/morphology and mechanical properties. These characteristics are determined by both dynamic actomyosin interactions as well as by the capacity of the actin cytoskeleton to rapidly depolymerize and repolymerize (Gerthoffer and Gunst, 2001; Yamin and Morgan, 2012). In this study, VSMC stiffness was measured by using the force required to indent the cell surface to a depth of ∼100–300 nm. In this case, the cell elasticity should be regarded as mainly representing the cortical stiffness of the cell’s stress fibers underlying the cell membrane. It is well established that the cortical cytoskeleton network physically connects to deeper cytoskeletal elements within the cytosol as well as to adhesion zones that regulate adhesion to the ECM (Fabry et al., 2011; Field and Lenart, 2011; Murrel and Gardel, 2012). Cortical stiffness is mechanically determined by cellular cortex elasticity and cortex tension, which is believed to be mostly governed by myosin-generated contractility (Reymann et al., 2012; Munjal and Lecuit, 2014). The changes in VSMC stiffness we observed in the presence of inhibition of MLCK are consistent with interpreting the alterations in stiffness as changes in the polymerization state of the cortical cytoskeleton.

Phenylephrine (PE) is another vasoconstrictor that has also been reported to induce cortical stiffening in VSMC without affecting focal adhesion size via FAK–ERK signaling. These data support a correlation between cellular cortical stiffness and cellular contractile state (Saphirstein et al., 2015). Together with the present result that pretreatment of ML-7 did not blunt KCl effects on cell stiffness and adhesion (Figure 5), these data further indicate calcium exerts its effect via calcium-related signaling pathway other than pathways downstream of actin-myosin interactions. Given previous findings from Hong et al. that cytoskeletal remodeling occurs in VMSCs following AII treatment (Hong et al., 2014), calcium appears to exert its effect on cell stiffness through changes in actin polymerization. Calcium-calmodulin-dependent protein kinase II (CaMKII) has been reported to regulate actin cytoskeletal assembly (Hoffman et al., 2013), which might suggest a possible mechanism of calcium effect. However, more experiments will be needed to demonstrate underlying mechanisms of involving cytoskeletal remodeling and calcium.

The responsiveness of VSMCs used in this study to AII confirmed the retention of characteristics expected of a contractile phenotype. This is important because cultured VSMCs carried in culture over many passages exhibit a transition from a contractile phenotype to a synthetic phenotype (Huber and Badylak, 2012; Chang et al., 2014). In our studies, only primary cell cultures were used without passage to minimize these phenotypic changes. Despite these precautions, it is not possible to completely prevent phenotypic shifting and this may account for why only 80% of the cells we studied were AII responsive (data are not shown).

Vascular smooth muscle cells cellular adhesion to FN was assessed by measuring the force required to produce rupture events between the FN-coated AFM probe adhering to the VSMC. This was quantified as the number of rupture events per force curve and the force that break adhesion bonds formed between VSMCs and FN on the AFM probe. An increase in detectable rupture events and/or force was interpreted as a change in integrin-mediated cell adhesion. Another possibility is that an increase in the number of rupture events induced by an agonist might be related to decrease in the cell surface area that resulted from mechanical crinkling or ruffling of the cell membrane, such that density of integrin per unit membrane area would be augmented and the number of detected adhesions would be also affected. However, pretreatment of VSMC with ML-7 to prevent any cell contraction did not change KCl-induced adhesion to FN (Figure 5). This evidence argues against a membrane contraction-induced change in integrin density. Also, in previous studies by Hong et al. (2012) increases in adhesion force were detected following AII suggested that the increase in adhesion was accompanied by changes in the molecular connections of the integrin to cytoskeletal components. This also argues against the argument of the change in membrane area.

The adhesion events and corresponding rupture forces measured by FN-coated AFM probes are due to interactions with α_5_β_1_ integrins (Sun et al., 2005; Trache et al., 2005). Thus, in this study, changes in adhesion are also likely α_5_β_1_ integrin-dependent. It has been demonstrated in migratory cells that integrin trafficking to and from the membrane can regulate focal adhesion size through assembly and/or disassembly resulting in spatial integrin redistribution of membrane integrins (Caswell et al., 2009). In our study, to assess whether there was a possible change of integrin expression on the membrane that could contribute to an increase in adhesion via the number of detectable rupture events, we coated spherical AFM probe with an anti-α_5_ antibody. The anti-α_5_ antibody that was selected shows the affinity for both active and inactive forms of the α_5_ integrin. We reasoned that if the AII was increasing the amount of integrin on the membrane then the anti-α_5_ antibody would show an increase in adhesion events following AII. Our results demonstrated that there was no change in adhesion to the anti-α_5_ antibody coated AFM probe following AII treatment (Figure 1). These data support the interpretation that AII-induced changes in adhesion to FN was occurring through a process of integrin activation rather than integrin turnover.

In summary, our studies show coordination of VSMC cortical stiffness and cell adhesion following contractile activation that is synchronized with changes in [Ca^2+^]_i_. ML-7 results further confirmed cell stiffness was related to contractile state since inhibition of MLCK reduced resting cell stiffness, but ML-7 did not inhibit KCl induced coordination of enhanced cortical stiffness and adhesion to FN. These observations suggest that calcium-induced cell stiffness and adhesion is parallel to the contractile activation pathway and not downstream of actin and myosin interaction. This information is an important advance in our understanding of the mechanisms underlying coordination of cell stiffness and adhesion to ECM. We propose this coordination is essential to provide the mechanical efficiency and ability of the VSMCs to appropriately adapt itself to mechanical changes brought about through intracellular contractile activation and likely extracellular changes in applied force. Increased vascular smooth muscle cell stiffness is a significant feature of aging and occurs in cardiovascular diseases, such as hypertension (Zhu et al., 2012; Sehgel et al., 2013). Further investigations into the underlying mechanisms governing both the cellular mechanics and adhesion of VSMC may lead to possible therapeutic targets that through the link between these pathways and vascular disease.

## Author Contributions

GM and MH conceived and designed the study, and analyzed and interpreted the data. ZS performed the experiments, analyzed the data, and interpreted the data. HH performed the experiments, analyzed the data, made figures and wrote the manuscript. GM edited the manuscript. All authors approved the final version of the manuscript.

## Conflict of Interest Statement

The authors declare that the research was conducted in the absence of any commercial or financial relationships that could be construed as a potential conflict of interest. The reviewers XP and XW and handling Editor declared their shared affiliation.

## Supplementary Material

Figure S1 Calcium level for ML-7 in Figure 5. Level of [Ca^2+^]_i_ summed across all of time points for the group of VSMCs before treatment, time points of 15 min of ML-7 (15 μM) and first three time points of after addition of KCl (60 μM) (*n* = 10, ^#^*p* < 0.05 compared to ML-7). Data were collected by FN-coated AFM at 0.1 Hz of indentation frequency and are presented as mean ± SEM.
