# Supplementary figures and images for: A Calcium Mediated Mechanism Coordinating Vascular Smooth Muscle Cell Adhesion During KCl Activation
Source: Front Physiol. 2018 Dec 18;9:1810. doi: 10.3389/fphys.2018.01810 (PMC6305448; doi:10.3389/fphys.2018.01810)

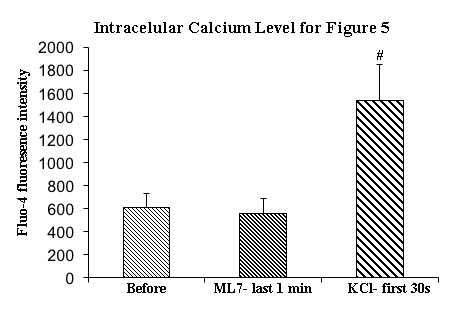

Supplement: Figure S1 — Calcium level for ML-7 in Figure 5. Level of [Ca2+]i summed across all of time points for the group of VSMCs before treatment, time points of 15 min of ML-7 (15 μM) and first three time points of after addition of KCl (60 μM) (n = 10, #p < 0.05 compared to ML-7). Data were collected by FN-coated AFM at 0.1 Hz of indentation frequency and are presented as mean ± SEM. [file Image_1.TIF]
